# Three strategies of transgenic manipulation for crop improvement

**DOI:** 10.3389/fpls.2022.948518

**Published:** 2022-07-22

**Authors:** Haoqiang Yu, Qingqing Yang, Fengling Fu, Wanchen Li

**Affiliations:** Maize Research Institute, Sichuan Agricultural University, Chengdu, China

**Keywords:** commercial release, heterologous expression, endogenous gene, exogenous gene, overexpression, suppressing expression, transgenic crop

## Abstract

Heterologous expression of exogenous genes, overexpression of endogenous genes, and suppressed expression of undesirable genes are the three strategies of transgenic manipulation for crop improvement. Up to 2020, most (227) of the singular transgenic events (265) of crops approved for commercial release worldwide have been developed by the first strategy. Thirty-eight of them have been transformed by synthetic sequences transcribing antisense or double-stranded RNAs and three by mutated copies for suppressed expression of undesirable genes (the third strategy). By the first and the third strategies, hundreds of transgenic events and thousands of varieties with significant improvement of resistance to herbicides and pesticides, as well as nutritional quality, have been developed and approved for commercial release. Their application has significantly decreased the use of synthetic pesticides and the cost of crop production and increased the yield of crops and the benefits to farmers. However, almost all the events overexpressing endogenous genes remain at the testing stage, except one for fertility restoration and another for pyramiding herbicide tolerance. The novel functions conferred by the heterologously expressing exogenous genes under the control of constitutive promoters are usually absent in the recipient crops themselves or perform in different pathways. However, the endogenous proteins encoded by the overexpressing endogenous genes are regulated in complex networks with functionally redundant and replaceable pathways and are difficult to confer the desirable phenotypes significantly. It is concluded that heterologous expression of exogenous genes and suppressed expression by RNA interference and clustered regularly interspaced short palindromic repeats-cas (CRISPR/Cas) of undesirable genes are superior to the overexpression of endogenous genes for transgenic improvement of crops.

## Introduction

### Breakthrough of crop improvement

To meet the food demand of the booming world population, the comprehensive requirements for yield, quality, and adaptability of crop cultivars are becoming more and more urgent (Barrett, 2021). Due to the limitation of genetic variation within nature or mutagenized populations of sexually compatible species, conventional approaches to crop improvement, such as systematic breeding, crossing breeding, and heterosis utilization, are laborious and time-consuming. However, transgenic technology surmounts hybridization barriers and utilizes the desirable genes from genetically distant species, to realize molecular design breeding to a certain extent (Raymond Park et al., 2011; Kamthan et al., 2016). It is thought that transgenic technology has been a revolutionary impact on crop improvement as a second Green Revolution, greatly improving the yield, quality, and adaptability of crops and making an important contribution to ensuring food security (Eckardt et al., 2009; Farre et al., 2010; Kamthan et al., 2016). Transgenic cultivars of crops are developed by cloning desirable genes, constructing expression vectors, genetic transformation of recipient crops, screening and identification of transformed lines, so as to improve the original undesirable traits or endow them with new beneficial traits (Raymond Park et al., 2011; Kamthan et al., 2016). In addition, transgenic technology is also used to modify or knock out the undesirable genes of crops to change their genetic characteristics and obtain the desirable phenotypes (Georges and Ray, 2017). After a safety assessment, transgenic cultivars with significant improvement in yield, quality, or adaptability are approved for commercial release.

### The rapid increase of transgenic crops

**FIGURE 1:**
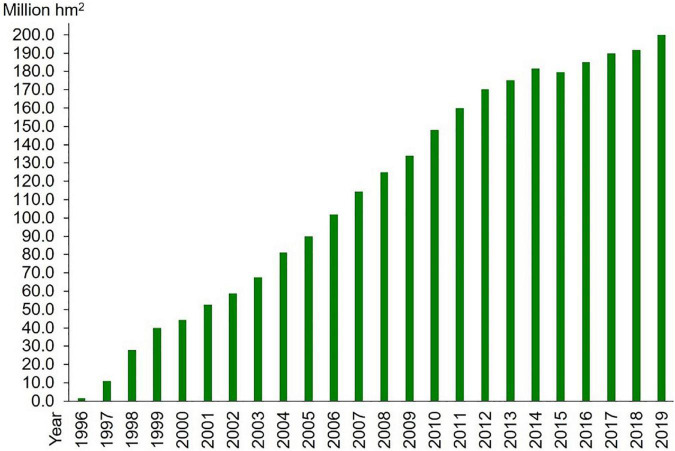
Commercialized acreage of transgenic crops.

The first transgenic plants were developed about four decades ago with traits like antibiotic and insect resistances (Bevan et al., 1983; Fraley et al., 1983; Herrera-Estrella et al., 1983; Murai et al., 1983). Since the approval of the transgenic tomato variety with delayed maturation for commercial release by the food and drug administration (FDA) after stringent scientific scrutiny and credible safety assessment in 1994 (Klee, 1993; Parrott et al., 2010; Giraldo et al., 2019), transgenic crops, like inset resistant cotton and maize, herbicide-resistant soybean and canola, have received marketing approval one after another (Padgette et al., 1995; Schuler et al., 1998; Bates et al., 2005), and transgenic technology has increased the pace of crop improvement to meet the requirements of biotic and abiotic resistance, higher yield, and nutritional value (Raymond Park et al., 2011). According to the survey carried out by the International Service for the Acquisition of Agri-Biotech Applications (ISAAA, 2022), the commercialized acreage of transgenic crops has straightly increased to 176.85 million hectares in the world by 2021 (Figure 1). This acreage distributes in more than 30 countries in all, including industrial and developing countries. Great profitability has been achieved by increasing yield and reducing input in pesticides, labor, and machinery (Naranjo, 2011; Raymond Park et al., 2011; Smyth et al., 2014).

## Achievements of three transgenic strategies

### Three transgenic strategies

Heterologous expression of exogenous genes, overexpression of endogenous genes, and suppressed expression of undesirable genes are the three strategies of transgenic manipulation for crop improvement. The first strategy is the transformation of crops by exogenous genes from genetically distant species. The second strategy is also proposed as cisgenesis and intragenesis by the transformation of endogenous genes from the same species or homologous genes from sexually compatible species, respectively, if no foreign DNA such as selectable marker gene and vector-backbone sequence is introduced into the cisgenes or intragenes by *in vitro* mutagenesis or other means (Rommens et al., 2007; Schouten and Jacobsen, 2008; Jacobsen and Schouten, 2009). These alternative concepts mitigate the public concerns about the biosafety of genetically modified (GM) crops developed by transgenesis (Schouten et al., 2006). The third strategy is to suppress the expression of undesirable endogenous or pathogenic and pest genes by the introduction of synthetic sequences transcribing antisense or double-stranded RNAs (Mamta and Rajam, 2017; Zhang J. et al., 2017; Hernández-Soto and Chacón-Cerdas, 2021), or to knock out them by clustered regularly interspaced short palindromic repeats-cas (CRISPR/Cas) technology as well as some other more complicated technologies of genome editing such as zinc finger nucleases (ZFNs), transcription activator-like effector nucleases (TALENs), and mega-nucleases (MNs) (Gaj et al., 2013; Jaganathan et al., 2018; Langner et al., 2018; Gao, 2021; Rasheed et al., 2021; Samaras et al., 2021; Sharma and Vakhlu, 2021; Turnbull et al., 2021). Especially CRISPR/Cas technology, as a simple, easy, and cost-effective tool of precise and straightforward genome-wide gene editing, has been developed as a potential strategy for crop improvement and helped much to mitigate the public’s negative perception of GM food crops (Naeem et al., 2020; Gao, 2021; Rasheed et al., 2021).

### Transgenic events approved for commercial release

According to the survey carried out by ISAAA (2022), most (227) of the singular transgenic events (265) approved for commercial release worldwide were developed by the first strategy: 210 by bacterial genes (Stalker et al., 1988; Ye et al., 2000; Paine et al., 2005; Castiglioni et al., 2008; Napier et al., 2019), 8 by exogenous genes from mold, algae, fungus, and yeast (Knutzon et al., 1998; Napier et al., 2019; Kinney et al., 2022), 5 by exogenous genes from sexually incompatible plant species (Song et al., 2003; Takagi et al., 2005; Preuss et al., 2012; Rice et al., 2014), and 2 by the mutant copies of the endogenous genes for enhancing herbicide tolerance (McNaughton et al., 2008; EFSA Panel on Genetically Modified Organisms [GMO]et al., 2018; Karthik et al., 2020), respectively (Table 1). Thirty-eight events were developed by the third strategy and transformed by synthetic sequences transcribing antisense or double-stranded RNAs for suppressed expression of undesirable endogenous genes of pathogens, pest insects, and recipient crops themselves (Chen et al., 2003; Davis and Ying, 2004; Tennant et al., 2005; Otani et al., 2007; Ilardi and Nicola-Negri, 2011; Aragao et al., 2013; Ramaseshadri et al., 2013; Carvalho et al., 2015; Orbegozo et al., 2016; Borah et al., 2018; Wu et al., 2018; Callahan et al., 2019; Chiozza et al., 2020). Only one event was transformed by endogenous genes for restoring male fertility (Unger et al., 2002) and another event for pyramiding herbicide tolerance. Of course, antibiotic or herbicide-resistant genes from bacteria were also introduced into almost all of these events as selection markers of transformant screening (Demaneche et al., 2008). By CRISPR/Cas technology, several events have developed, skipped regulation of government, and entered the market because of their safety assurance, and some more events have been in the pipeline of safety assessment (Hartung and Schiemann, 2014; Waltz, 2016a,b; Wolt et al., 2016; Faure and Napier, 2018; Jaganathan et al., 2018; Langner et al., 2018; Samaras et al., 2021).

**TABLE 1:** Singular transgenic events approved for commercial release.

GM crop	GM trait	Event	Bacterial	Distant species	Plant			RNAi
					Exogenous	Mutant	Endogenous	
Alfalfa	Herbicide tolerance	2	2					
	Quality improvement	4						4
Bean	Viral resistance	1						1
Canola	Herbicide tolerance	23	23					
	Quality improvement	9	2	7	1			
Cotton	Herbicide tolerance	28	28		3			
	Insect resistance	26	27		1			
	Quality improvement	1						1
Cowpea	Insect resistance	1	1					
Eggplant	Insect resistance	1	1					
Flax	Herbicide tolerance	1	1					
Maize	Herbicide tolerance	18	16			3	1	
	Insect resistance	7	7					
	Herbicide/insect tolerance	18	18					
	Drought tolerance	1	1					
	Quality improvement	5	3	1	1			
	Male sterility	3	3					
	Fertility restoration	1					1	
Melon	Delayed maturation	2	2					
Soybean	Herbicide tolerance	18	17		1	3		
	Insect resistance	2	2					
	Herbicide/insect tolerance	2	2					
	Herbicide tolerance/growth regulation	1			1			
	Drought tolerance	1			1			
	Quality improvement	4			1	2		1
Papaya	Viral resistance	4						4
Plum	Viral resistance	1						1
Potato	Herbicide tolerance	4	4					
	Insect resistance	29	29					
	Viral resistance	15						15
Rice	Herbicide tolerance	3	3					
	Insect resistance	3	3					
	Quality improvement	2	2		1		1	
Squash	Viral resistance	2						2
Sugar beet	Herbicide tolerance	3	3					
Sugarcane	Insect resistance	3	3					
	Drought tolerance	3	3					
Sweet pepper	Viral resistance	1						1
Tobacco	Herbicide tolerance	1	1					
	Quality improvement	1						1
Tomato	Insect resistance	1	1					
	Viral resistance	1						1
	Delayed maturation	9	2			1		6
Wheat	Herbicide tolerance	1	1					
Total		265	210	8	5 (+6)	3 (+6)	2 (+1)	38
Technological strategy			First				Second	Third
			227 (+12)				2 (+1)	38

Distant species include mold, algae, fungus, and yeast. RNAi was triggered by antisense and double-stranded RNA described by the introduced synthetic DNA sequence. The gray background indicates the stacked genes. The numbers of the events introduced stacked genes were put in the brackets. All the information is from ISAAA (2022).

### Great achievements of heterologous expression of exogenous genes

**FIGURE 2:**
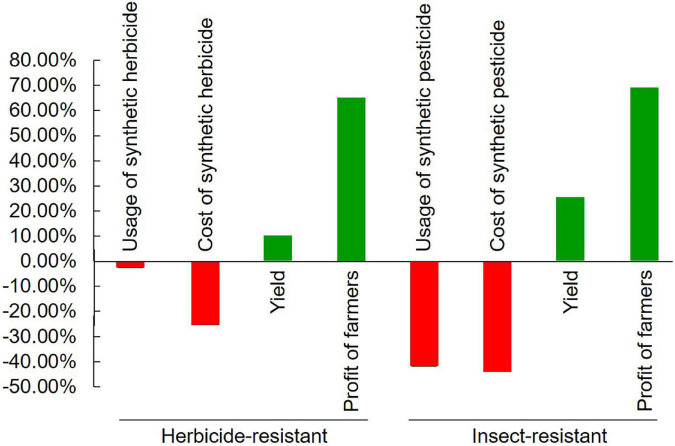
Benefits of transgenic herbicide- and insect-resistant crops.

Weeds burden plant growth as they compete for space, sunlight, and soil nutrients leading to 25–80% yield losses (Awan et al., 2015; Ramachandra et al., 2016). The application of synthetic herbicides is an effective approach to control weeds but causes great waste of resources, and serious problems of environmental pollution and food safety (Vandenberg et al., 2017; Panthi et al., 2019). GM varieties transformed by herbicide-resistant genes give the feasibility to combat weeds and thus help in the safety of the crops without major yield losses (Benbrook, 2016; Ramachandra et al., 2016). Glyphosate [*N*-(phosphonomethyl) glycine] is a widely used broad-spectrum herbicide that controls weeds by inhibiting the 5-enolpyruvylshikimate-3-phosphate synthase (EPSPS) enzyme and interfering with the shikimate biosynthesis pathway (Funke et al., 2006). However, this non-selective herbicide also damages the crops. The current strategy is to improve glyphosate resistance in crops by the transformation of the *EPSPS*, *GAT*, and *Goxv* genes that encode for an insensitive form of EPSPS of acetyltransferase and glyphosate oxidase, respectively (Block et al., 1987; Padgette et al., 1995; Castle et al., 2004; McNaughton et al., 2008; Yan et al., 2011; Awan et al., 2015; Chhapekar et al., 2015; Guo et al., 2015; Liang et al., 2017; Zhang X. B. et al., 2017; Yi et al., 2018). The other widely used synthetic herbicides are gluphosinate and bialaphos, which inhibit the activity of glutamine synthetase and thus block all nitrogen assimilation into the plant. Tolerant varieties are developed by the transformation of the genes *PAT* and *Bar* that encode for phosphinothricin acetyltransferase (PAT), which detoxifies the herbicides (Hérouet et al., 2005; Herman et al., 2013; Cegielska-Taras et al., 2008; EFSA Panel on Genetically Modified Organisms [GMO] et al., 2020a,b). Bromoxynil (3,5-dibromo 4-hydroxybenzonitrile) and other oxynil herbicides inhibit photosynthesis by blocking electron flow during the light reaction, causing the production of reactive oxygen species (ROS), destruction of cell membranes, inhibition of chlorophyll formation and death. Resistance is conferred by the *bxn* gene that encodes a nitrilase enzyme that detoxifies the herbicide (Stalker et al., 1988). All these herbicide-resistant genes are derived from various species of soil bacteria *Agrobacterium*, *Pseudomonas*, *Streptomyces*, *Bacillus*, *Ochrobactrum*, and *Klebsiella* (Block et al., 1987; Thompson et al., 1987; Stalker et al., 1988; Castle et al., 2004; Siehl et al., 2007; Yan et al., 2011). Only very few of them are transformed by an herbicide-resistant mutant of exogenous or endogenous plant *EPSPS* genes (Table 1; McNaughton et al., 2008; EFSA Panel on Genetically Modified Organisms [GMO]et al., 2018; Karthik et al., 2020). From these transgenic events, 4 alfalfa, 52 canola, 65 cotton, 345 maize, 4 potato, 3 rice, 52 soybean, 2 sugar beet, 1 tobacco, and 1 wheat cultivars have been developed and approved for commercial release^1^. A meta-analysis shows that the application of these transgenic herbicide-resistant varieties reduces the use of synthetic herbicide by 2.43% and the cost of herbicide by 25.29%, increases the yield of crops by 9.29%, and benefits farmers by 64.29% (Figure 2; Klumper and Qaim, 2014). However, great attention should be paid to the potential environment and agronomic impact caused by intraspecific gene flow from the transgenic herbicide-resistant varieties to weeds or their non-GM counterparts, especially in partly cross-pollinated canola and other crops of the crucifer family (Légère, 2005; Bonny, 2016; Sharkey et al., 2021). This problem should be seriously solved by the choice of suitable cultivars and certified seeds as well as by weed and soil management (Devos et al., 2004; Hüsken and Dietz-Pfeilstetter, 2007). The potential CRISPR/Cas technology should play important role in combating this problem (Hussain et al., 2021).

Insect pests used to be the major biotic stress that caused a serious reduction in crop productivity globally (Oerke, 2006; Douglas, 2018). The extensive application of chemical pesticides not only increased production costs but also caused severe environmental pollution (Aktar et al., 2009; Birkett and Pickett, 2014). Transgenic insect-resistant crops (mainly cotton, maize, and soybean) have made a beneficial and eco-friendly impact on crop production (Matten and Reynolds, 2003; Gatehouse et al., 2011; Blanco, 2012; Rocha-Munive et al., 2018). The majority (68) of the transgenic insect-resistant events (70) are developed by heterologous expression of the insecticidal genes *Cry* (δ-endotoxin) from different strains of soil bacterium *Bacillus thuringiensis* (Ghareyazie et al., 1997), except for 1 maize, 2 poplar, and 2 cotton events simultaneously transformed by vegetative insecticidal protein genes *vip3*, *CpTI*, and *API*, as well as double-stranded RNA transcript of gene *Snf7* from western corn rootworm (*Diabrotica virgifera*), respectively, for pyramiding broad resistance (Table 1; Xie et al., 1997; Hu et al., 2001; Cui et al., 2011; Ramaseshadri et al., 2013). From these events, 59 cotton, 1 cowpea, 1 eggplant, 341 maize, 3 poplar, 30 potato, 3 rice, 6 soybean, 3 sugarcane, and 1 tomato cultivars resistant to lepidopteran (246), coleopteran (156), hemipteran (1), as well as multiple insects (36), respectively, have been developed and approved for commercial release (ISAAA, 2022). The majority of published studies on transgenic cotton performance have documented the decrease in insecticide application, and the increase in yield and benefits in developed and developing countries (Showalter et al., 2009). A meta-analysis shows that the application of insecticidal transgenic crops has decreased the use of synthetic pesticides by 41.67% and the cost of pesticides by 43.43%, increased the yield of crops by 24.85%, and benefited farmers by 68.78% (Figure 2; Klumper and Qaim, 2014). This analysis is confirmed by an actual survey on the application of transgenic insect-resistant cotton in China (Pray et al., 2001).

Vitamin A deficiency is common in children in developing countries who rely on rice as a staple food (Cabezuelo et al., 2020). Transgenic “Golden” rice and potato heterologously expressing three bacterial genes (*CrtB*, *CrtI*, and *CrtY*) encoding phytoene synthase, phytoene desaturase, and lycopene β-cyclase, respectively, for enhancing β-carotene synthesis and accumulation have been developed and approved for commercial release, and directly used as food in the United States, Canada, New Zealand, Australia, Nigeria, Kenya, and the Philippines (Table 1; Ye et al., 2000; Paine et al., 2005; Chitchumroonchokchai et al., 2017; Napier et al., 2019), although endogenous orthologs of these genes are present in the genomes of rice and potato (Thorup et al., 2000; Koc et al., 2015; Banakar et al., 2020; Yang et al., 2021).

Canola (*Brassica napus*) is a high-yield oil crop. However, the quality of its oil is required to be improved to decrease the high proportion of saturated fatty acids. Genetic modification by introducing desaturase genes and desaturase-related genes is an effective approach (Napier et al., 2019). Although these genes are present in the genome of canola itself (Xue et al., 2018), all the nine events approved for commercial release (ISAAA, 2022) have been transformed by stacked exogenous genes from mold, algae, fungus, and yeast (Table 1; Knutzon et al., 1998; Napier et al., 2019; Kinney et al., 2022).

### Overexpression of endogenous genes remains at the testing stage

By the strategy of overexpression of endogenous genes, numerous transgenic events have also been developed and their target phenotypes reported to be enhanced. For example, drought is one of the most significant constraints on crop production (Cohen et al., 2021). A lot of literatures have documented the improvement of drought tolerance of transgenic crops overexpressing endogenous or homologous genes encoding function proteins related to osmotic protectants, membrane stabilization, detoxification and transport, such as SOD (superoxide dismutase), VP1 (vacuolar proton-pumping pyrophosphatase), BADH (betaine aldehyde dehydrogenase), P5CS (Δ1-pyrroline-5-carboxylate synthetase), LEA (late embryogenesis-abundant proteins), and FER (ferritin), as well as transcription factors and signaling molecules, such as DREB (dehydration-responsive element binding), ABF [abscisic acid (ABA)-responsive elements binding factor], AP2/ERF (ethylene response factor), bZIP (basic leucine zipper), MYB, MYC, NAC (no apical meristem), ZFP (zinc finger protein), HD-Zip (homeodomain-leucine zipper), WRKY, NF (nuclear factor), HRD, and HYR (higher yield rice) (Kasuga et al., 1999; Nelson et al., 2007; Xiao et al., 2007; Century et al., 2008; Huang et al., 2009; Wu et al., 2009; Tran et al., 2010; Varshney et al., 2011; Xue et al., 2011; Datta et al., 2012; Schilling et al., 2017; Bi et al., 2018; Gao et al., 2018; Yang et al., 2018; Sarkar et al., 2019; Wei et al., 2019). However, the vast majority of these efforts have still remained at the testing stage. Of the five singular transgenic events approved for commercial release (Table 1; ISAAA, 2022), one has been transformed by the cold shock protein gene *CspB* from *B. subtilis* and three by the choline dehydrogenase genes *BetA* from *Escherichia coli* and *Rhizobium meliloti*, respectively, although the cold shock proteins are also found in many eukaryotic species (Castiglioni et al., 2008; Tollefson, 2011). Only one has been transformed by the exogenous transcription factor gene *Hahb-4* from sexually incompatible sunflower (*Helianthus annuus*) (Ribichich et al., 2020).

### RNA interference is effective for suppressing expression of undesirable genes

Plant diseases reduce crop yield and quality and bring huge economic losses (Gimenez et al., 2018). Transgenic technology has been employed to battle against a wide range of plant pathogens (Wally and Punja, 2010; Kamthan et al., 2016). Similar to drought tolerance, the vast majority of the transgenic events overexpressing endogenous disease-resistant genes or homologous disease-resistant genes from sexually incompatible species remain at the testing stage (Anand et al., 2003; Zhao et al., 2005; Yang et al., 2008; Zhou et al., 2009). Twenty-five of the 29 approved events have been developed by the third strategy and transformed with synthetic DNA sequences to transcribe antisense or double-stranded RNAs for the interference of disease viruses (Chen et al., 2003; Davis and Ying, 2004; Tennant et al., 2005; Aragao et al., 2013; Carvalho et al., 2015; Borah et al., 2018; Wu et al., 2018; Callahan et al., 2019; Chiozza et al., 2020), and only the other four potato events are transformed with exogenous genes of pathogenesis-related proteins from distant species of the nightshade family (*Solanum bulbocastanum* and *Solanum venturii*) (Table 1; Halterman et al., 2008; Foster et al., 2009). RNA interference (RNAi) triggered by antisense or double-stranded RNAs described by transformed synthetic DNA sequences is a versatile, effective, safe, and eco-friendly technology for crop protection against viruses and other pathogens as well as insect pests, and delaying maturation of fruits with positive economic, environmental, and human health implications (Klee, 1993; Taning et al., 2020; Giudice et al., 2021; Hernández-Soto and Chacón-Cerdas, 2021).

### Application of clustered regularly interspaced short palindromic repeats/Cas9 for crop improvement

Since its first discovery in *E. coli* (Ishino et al., 1987), CRISPR/Cas has been developed as a simple, easy, and cost-effective tool for precise and straightforward genome-wide gene editing (Gaj et al., 2013; Tang et al., 2017; Molla and Yang, 2019; Molla et al., 2020, 2021; Sharma and Vakhlu, 2021). Unlike ZFNs, TALENs, and MNs, CRISPR/Cas could be used to modify any genomic sequences, thereby providing a simple, easy, and cost-effective means of precise and straightforward genome-wide gene editing (Gaj et al., 2013; Tang et al., 2017; Gao, 2021; Leibowitz et al., 2021; Rasheed et al., 2021; Sharma and Vakhlu, 2021; Turnbull et al., 2021). However, In the beginning, most of the studies focused more on the concept proofing of the CRISPR/Cas system (Nekrasov et al., 2013; Shan et al., 2013; Xie and Yang, 2013; Zhang et al., 2014; Lawrenson et al., 2015; Xie et al., 2015; Hu et al., 2016; Malnoy et al., 2016; Zhu et al., 2016; Li et al., 2017; Shimatani et al., 2017). Although many attempts have been made to improve the yield, quality, and biotic and abiotic tolerance of different crops (Xie and Yang, 2013; Liang et al., 2014; Wang et al., 2014, 2021; Zhang et al., 2014, 2018, 2020; Fang and Tyler, 2016; Li et al., 2016, 2017, 2020; Zaidi et al., 2016; Zhu et al., 2016; Shi et al., 2017; Shimatani et al., 2017; Yang et al., 2017; Kim et al., 2018; Okuzaki et al., 2018; Shen et al., 2018; Usman et al., 2020, 2021; Zeng et al., 2020; Monsur et al., 2021), only very few events have been in the pipeline of safety assessment up to now (Waltz, 2016a,b). In recent years, several techniques, such as high attractive sgRNA, high fidelity Cas9, and transformant screening, have been developed to reduce the probable off-target effects caused by the imperfect matches with gRNA and the unpredictable efficiency among different DNA target sites and PAM (Naeem et al., 2020; Leibowitz et al., 2021). CRISPR/Cas has been improved as the most promising tool for crop improvement (Gao, 2021; Rasheed et al., 2021; Turnbull et al., 2021) and applied to improve yield, quality, and biotic and abiotic tolerance (Wang et al., 2014, 2021; Fang and Tyler, 2016; Li et al., 2016, 2017, 2020; Malnoy et al., 2016; Zaidi et al., 2016; Braatz et al., 2017; Shi et al., 2017; Yang et al., 2017; Okuzaki et al., 2018; Shen et al., 2018; Zhang et al., 2018, 2020; Usman et al., 2020, 2021; Zeng et al., 2020; Monsur et al., 2021). Up to now, several events have been developed and skipped regulation of government and entered the market because of their safety assurance, and some more events have been in the pipeline of safety assessment (Hartung and Schiemann, 2014; Waltz, 2016a,b; Wolt et al., 2016; Faure and Napier, 2018; Jaganathan et al., 2018; Langner et al., 2018; Samaras et al., 2021).

## Superiority of heterologous expression of exogenous genes

### Novel functions conferred by exogenous genes are less regulated by endogenous pathways

The functions conferred by the heterologously expressing exogenous genes are usually novel in the recipient crops themselves, such as herbicide and insect resistance in the released events of transgenic cotton, maize, soybean, and alfalfa (Cui et al., 2011; Awan et al., 2015; Chhapekar et al., 2015; Guo et al., 2015; Liang et al., 2017; Yi et al., 2018), or perform in different pathways, such as the synthesis of β-carotene and unsaturated fatty acids in the released events of transgenic rice and canola (Knutzon et al., 1998; Ye et al., 2000; Paine et al., 2005; Wan et al., 2017). In the vast majority of the above transgenic events, the exogenous genes are promoted by the constitutive promoters (Paine et al., 2005; Cui et al., 2011; Awan et al., 2015; Chhapekar et al., 2015; Guo et al., 2015; Liang et al., 2017; Zhang J. et al., 2017; Yi et al., 2018). Therefore, their expression is usually not regulated on the transcriptional level, although some other factors such as genetic background and growth stage of the recipient cultivars, and environmental conditions may affect their expression by several folds (Adamczyk and Meredith, 2004; Showalter et al., 2009; Poongothai et al., 2010; Chen et al., 2019). In case of the transgenic events are developed by the introduction of distant prokaryotic genes, possible codon usage bias is usually overcome by codon optimization of the transgene sequences (Siehl et al., 2007; Liu, 2009; Yan et al., 2011; Chhapekar et al., 2015; Liang et al., 2017; Yi et al., 2018). The investigations in transgenic insecticidal cotton (*r* = 0.762, *p* < 0.001) and rice (*r* = 0.742, *p* < 0.01) show that the accumulation of the Cry protein in leaves is non-linearly correlated with the heterologous transcription levels of the exogenous *Cry* genes, although varying with growth and development (Adamczyk and Sumerford, 2001; Adamczyk et al., 2001, 2009; Zhang et al., 2016). Adamczyk and Meredith (2004) suggest that a small number of endogenous genetic factors control the accumulation of the Cry protein in transgenic cotton. Therefore, these exogenous proteins usually diverge from the endogenous metabolism pathways of the recipient crops (Sanahuja et al., 2011; Palma et al., 2014; Melo et al., 2016). The resistance conferred by these prokaryotic toxins is not easy to be overcome by the evolution of the pest insects. Instead, it can be augmented and complemented by pyramiding broad resistance expressing different combinations of insecticidal genes with different insecticidal mechanisms, or silencing the housekeeping genes of pest insects by RNAi and CRISPR/Cas technologies (Bates et al., 2005; Carriere et al., 2015; Katta et al., 2020; Talakayala et al., 2020). In agricultural practice, integrated pest management is still necessary to control the non-targeted pests of Cry protein (Naranjo, 2011; Downes et al., 2017).

### Endogenous proteins are regulated in complex networks

Almost all biochemical reactions are reversible, and most of them are regulated in complex networks (Alberty, 2002; Fiehn and Weckwerth, 2003). One of the fundamental predictions of metabolic control theory is that, while any step in a pathway can be made to control flux if the step is blocked, increasing the activity of an enzyme may not necessarily result in increased flux through the reaction it catalyzes (Kacser and Burns, 1995). For example, plant lipids are a complex mixture of several hundreds of triacylglycerol fatty acids (Dormann, 2021). The relative content and saturation degree of these fatty acids determine the functional, sensory and nutritional value of the oil. Their synthesis metabolism is well regulated in a complex network among alternative pathways across multiple subcellular compartments (King et al., 2015; Chapman and Feussner, 2016; Haslam et al., 2016; Wan et al., 2017). In soybean cotyledon, the activity of stearoyl-acyl carrier protein (ACP) desaturase is excess and thus overexpression of an endogenous stearoyl-ACP desaturase gene does not result in any changes in the accumulation and proportion of fatty acids (Kinney, 1996; Voelker and Kinney, 2001). However, the synthesis pathway can be rationally modified by the introduction of exogenous genes from distant species to produce novel fatty acids of high value that are absent or typically found at low levels in oil crops (Voelker and Kinney, 2001; Thelen and Ohlrogge, 2002; Haslam et al., 2016). All the four transgenic canola events approved for commercial release (ISAAA, 2022) are introduced with stacked exogenous genes from distant species of bacterium, mold, yeast, algae, fungus, moss, and amastigote (Knutzon et al., 1998; Napier et al., 2019).

Another example is the transgenic improvement of drought tolerance. Drought tolerance of plants is mediated by signal transduction ionic and osmotic homeostasis, detoxification, and growth pathways. The ionic aspect is signaled *via* the SOS pathway where a calcium-responsive SOS3-SOS2 protein kinase complex controls the expression and activity of ion transporters such as SOS1. Osmotic stress activates several protein kinases including mitogen-activated kinases, which may mediate osmotic homeostasis and/or detoxification responses. A number of phospholipid systems are activated by osmotic stress, generating a diverse array of messenger molecules, some of which may function upstream of the osmotic stress-activated protein kinases. The phytohormone ABA plays a crucial role in plant growth and development, especially in response to abiotic stresses. The endogenous ABA level is controlled by complex regulatory mechanisms involving biosynthesis, catabolism, transport, and signal transduction pathways. This complex regulatory network responds to abiotic stresses at multiple levels, including transcription, translation, and post-translational regulation of tolerance-related genes (Zhu, 2002, 2016; Cutler et al., 2010; Dong et al., 2015). After perception by proteins of the PYR/PYL/RCAR family, the ABA-bound PYR/PYL/RCARs interact with clade A protein phosphatases type 2Cs (PP2Cs) and prevent them from inhibiting the sucrose non-fermenting 1-related protein kinase 2s (SnRK2s) (Fujii et al., 2009; Ma et al., 2009; Park et al., 2009). The activated SnRK2s induce ABA-responsive gene expression by phosphorylating transcription factors such as ABA-responsive element-binding factors (ABFs) and regulate many other processes through phosphorylating other substrates (Umezawa et al., 2013; Wang et al., 2013). Conversely, recent researches show that the PYR/PYL/RCAR receptors themselves are regulated by other pathways (Yu et al., 2020), and even they repress the activity of ABA-independent SnRK2s (Zhao et al., 2018). In the maize genome, there are 13, 16, and 11 members in the ZmPYL family, clade A of the ZmPP2C family, and the ZmSnRK2 family, respectively. Therefore, the possible alternative pathways have as many as 2288 (13 × 16 × 11) from the ZmPYLs through the ZmPP2Cs to the ZmSnRK2s (Wang et al., 2018). Most of these pathways are functionally redundant and replaceable, so overexpression of the endogenous genes in any of these 2288 pathways is difficult to cause a significant improvement for tolerant phenotypes (Zhu, 2016). Their transformed lines remain at the testing stage (Hu et al., 2010; Xiang et al., 2017; Bhatnagar et al., 2020; Wang et al., 2020). Moreover, transformation may generate completely new interactions between the transgenes making them function differently from what is expected. Possible negative interactions between the desired phenotypes and other traits should be accounted (Khan et al., 2019). For example, the barley and wheat transformants of wheat transcription factor gene *HD-ZipI* (homeodomain-leucine zipper) and the tobacco transformant of maize DREB gene *ZmDREB4.1* showed improved resistance to drought but also exhibited an undesirable reduction of biomass and yield (Kovalchuk et al., 2016; Li et al., 2018; Yang et al., 2018). In many studies, the tolerance of transgenic events was evaluated by pot experiments in a greenhouse, which was different from the response of plants to water deficit in a gradual manner under natural conditions (Ortiz et al., 2007; Passioura, 2012; Pierre et al., 2012). A review by Cattivelli et al. (2008) of improvements in drought tolerance considers the new insights into the complexity of plant mechanisms enabled by genomics, but there is still a large gap between yields in optimal and stress conditions.

## Conclusion

(1) The vast majority of the singular transgenic events approved for commercial release worldwide are transformed by genetically distant exogenous genes. The heterologous expression of these genes improves the resistance of crops to herbicides and pesticides, as well as the nutritional quality. Their application significantly decreases the use of synthetic herbicides and pesticides, reduces cost, increases the yield of crops, and benefits farmers. The novel functions conferred by these genes under the control of constitutive promoters therefore, over, are usually absent in the recipient crops themselves or perform in different pathways. On the other hand, the functions of endogenous proteins are redundant and replaceable in complex networks. Therefore, overexpression of endogenous genes is difficult to cause a significant improvement of phenotypes as the heterologous expression of exogenous genes (Table 2).
(2) RNAi triggered by antisense or double-stranded RNAs described by transformed synthetic DNA sequences is a versatile, effective, safe, and eco-friendly technology for crop protection against viruses and other pathogens as well as insect pests, and delaying maturation of fruits with positive economic, environmental, and human health implications (Table 2).
(3) CRISPR/Cas has developed as the most promising tool for crop improvement. Up to now, several events developed have skipped regulation of government and entered the market because of their safety assurance, and some more events have been in the pipeline of safety assessment (Table 2).

**TABLE 2:** Advantages and limitations of three transgenic strategies.

	The first strategy	The second strategy	The third strategy	
			RNAi	CRISPR/Cas
Transformed sequence	Genetically distant genes	Endogenous genes	Antisense or double-stranded DNAs	CRISPR and Cas9
Phenotype	Conferring novel phenotypes	Enhancing desirable phenotypes	Suppressing undesirable phenotypes	Modifying phenotypes
Regulation	Novel proteins functioning in diverse pathways	Endogenous proteins regulated in complex networks	Suppressing synthesis of target proteins	Suppressing or modifying synthesis of target proteins
Approved event	227	2	38	1

## Author contributions

HY drafted the manuscript. QY was responsible for data statistics. FF and WL conceived and supervised the research. All authors interpreted and discussed the data.

## Conflict of interest

The authors declare that the research was conducted in the absence of any commercial or financial relationships that could be construed as a potential conflict of interest.

## Publisher’s note

All claims expressed in this article are solely those of the authors and do not necessarily represent those of their affiliated organizations, or those of the publisher, the editors and the reviewers. Any product that may be evaluated in this article, or claim that may be made by its manufacturer, is not guaranteed or endorsed by the publisher.

## Footnotes

1
https://www.isaaa.org/gmapprovaldatabase

## References

[B1] Adamczyk J. J. Hardee D. D. Adams L. C. Sumerford D. V. (2001). Correlating differences in larval survival and development of bollworms (Lepidoptera: Noctuidae) and fall armyworms (Lepidoptera: Noctuidae) to differential expression of Cry1A(c) δ-endotoxin in various plant parts among commercial cultivars of transgenic *Bacillus thuringiensis* cotton. *J. Econ. Entomol.* 94 284–290. 10.1603/0022-0493-94.1.284 11233127

[B2] Adamczyk J. J. Meredith W. R. (2004). Genetic basis for variability of Cry1Ac expression among commercial transgenic *Bacillus thuringiensis* (Bt) cotton cultivars in the United States. *J. Cotton Sci.* 8 17–23.

[B3] Adamczyk J. J. Perera O. Meredith W. R. (2009). Production of mRNA from the Cry1Ac transgene differs among Bollgard lines which correlates to the level of subsequent protein. *Transgenic Res.* 18 143–149. 10.1007/s11248-008-9198-z 18594999

[B4] Adamczyk J. J. Sumerford D. V. (2001). Potential factors impacting season-long expression of Cry1Ac in 13 commercial varieties of Bollgard cotton. *J. Insect Sci.* 1:13. 15455073 PMC355897

[B5] Aktar W. Sengupta D. Chowdhur Y. A. (2009). Impact of pesticides use in agriculture: their benefits and hazards. *Interdiscip. Toxicol.* 2 1–12. 10.2478/v10102-009-0001-7 21217838 PMC2984095

[B6] Alberty R. A. (2002). Thermodynamics of systems of biochemical reactions. *J. Theor. Biol.* 215 491–501. 10.1006/jtbi.2001.2516 12069492

[B7] Anand A. Zhou T. Trick H. N. Gill B. S. Bockus W. W. Muthukrishnan S. (2003). Greenhouse and field testing of transgenic wheat plants stably expressing genes for thaumatin-like protein, chitinase and glucanase against *Fusarium graminearum*. *J. Exp. Bot.* 54 1101–1111. 10.1093/jxb/erg110 12598580

[B8] Aragao F. J. L. Nogueira E. O. P. L. Tinoco M. L. P. Faria J. C. (2013). Molecular characterization of the first commercial transgenic common bean immune to the bean golden mosaic virus. *J. Biotechnol.* 166 42–50. 10.1016/j.jbiotec.2013.04.009 23639387

[B9] Awan M. F. Abass A. Muzaffar A. Ali A. Tabassum B. Rao A. Q. (2015). Transformation of insect and herbicide resistance genes in cotton (*Gossypium hirsutum* L.). *J. Agri. Sci. Technol.* 17 287–298.

[B10] Banakar R. Schubert M. Collingwood M. Vakulskas C. Eggenberger A. L. Wang K. (2020). Comparison of CRISPR-Cas9/Cas12a ribonucleoprotein complexes for genome editing efficiency in the rice phytoene desaturase (OsPDS) gene. *Rice* 13:4. 10.1186/s12284-019-0365-z 31965382 PMC6973557

[B11] Barrett C. B. (2021). Overcoming global food security challenges through science and solidarity. *Am. J. Agri. Econ.* 103 422–447. 10.1111/ajae.12160

[B12] Bates S. Zhao J. Z. Roush R. T. Shelton A. M. (2005). Insect resistance management in GM crops: past, present and future. *Nat. Biotechnol.* 23 57–62. 10.1038/nbt1056 15637622

[B13] Benbrook C. M. (2016). Trends in glyphosate herbicide use in the United States and globally. *Environ. Sci. Eur.* 28:3. 10.1186/s12302-016-0070-0 27752438 PMC5044953

[B14] Bevan M. W. Flavell R. B. Chilton M. D. (1983). A chimeric antibiotic resistance gene as a selectable marker for plant cell transformation. *Nature* 304 184–187. 10.1038/304184a0 1422041

[B15] Bhatnagar N. Kim R. Han S. Song J. Lee G. S. Lee S. (2020). Ectopic expression of OsPYL/RCAR7, an ABA receptor having low signaling activity, improves drought tolerance without growth defects in rice. *Inter. J. Mol. Sci.* 21:4163. 10.3390/ijms21114163 32545174 PMC7312952

[B16] Bi H. Shi J. Kovalchuk N. Luang S. Bazanova N. Chirkova L. (2018). Overexpression of the TaSHN1 transcription factor in bread wheat leads to leaf surface modifications, improved drought tolerance, and no yield penalty under controlled growth conditions. *Plant Cell Environ.* 41 2549–2566. 10.1111/pce.13339 29761511

[B17] Birkett M. A. Pickett J. A. (2014). Prospects of genetic engineering for robust insect resistance. *Curr. Opin. Plant Biol.* 19 59–67. 10.1016/j.pbi.2014.03.009 24747775

[B18] Blanco C. A. (2012). Heliothis virescens and Bt cotton in the United States. *GM Crop Food* 3 201–212. 10.4161/gmcr.21439 22892654

[B19] Block M. D. Bottermann J. Vandewiele M. Dockx T. Thoen C. Gossele V. (1987). Engineering herbicide resistance into plants by expression of a detoxifying enzyme. *EMBO J.* 6 2513–2518. 10.1002/j.1460-2075.1987.tb02537.x 16453789 PMC553667

[B20] Bonny S. (2016). Genetically modified herbicide-tolerant crops, weeds, and herbicides: overview and impact. *Environ. Manag.* 57 31–48. 10.1007/s00267-015-0589-7 26296738

[B21] Borah M. Berbati M. Reppa C. Holeva M. Nath P. D. Voloudakis A. (2018). RNA-based vaccination of Bhut Jolokia pepper (*Capsicum chinense* Jacq.) against cucumber mosaic virus. *Virusdisease* 29 207–211. 10.1007/s13337-018-0452-6 29911155 PMC6003052

[B22] Braatz J. Harloff H. J. Mascher M. Stein N. Himmelbach A. Jung C. (2017). CRISPR-Cas9 targeted mutagenesis leads to simultaneous modification of different homoeologous gene copies in polyploid oilseed rape (*Brassica napus*). *Plant Physiol.* 174 935–942. 10.1104/pp.17.00426 28584067 PMC5462057

[B23] Cabezuelo M. T. Zaragoza R. Barber T. Vina J. R. (2020). Role of vitamin A in mammary gland development and lactation. *Nutrients* 12:80. 10.3390/nu12010080 31892157 PMC7019238

[B24] Callahan A. M. Dardick C. D. Scorza R. (2019). Multilocation comparison of fruit composition for ‘HoneySweet’, an RNAi based plum pox virus resistant plum. *PLoS One* 14:e0213993. 10.1371/journal.pone.0213993 30901368 PMC6430400

[B25] Carriere Y. Crickmore N. Tabashnik B. E. (2015). Optimizing pyramided transgenic Bt crops for sustainable pest management. *Nat. Biotechnol.* 33 161–168. 10.1038/nbt.3099 25599179

[B26] Carvalho J. L. De Oliveira Santos J. Conte C. Pacheco S. Nogueira E. O. Souza T. L. (2015). Comparative analysis of nutritional compositions of transgenic RNAi-mediated virus-resistant bean (event EMB-PV051-1) with its non-transgenic counterpart. *Transgenic Res.* 24 813–819. 10.1007/s11248-015-9877-5 25894661

[B27] Castiglioni P. Warner D. Bensen R. R. Anstrom D. D. Harrison J. Stoecker M. (2008). Bacterial RNA chaperones confer abiotic stress tolerance in plants and improved grain yield in maize under water-limited conditions. *Plant Physiol.* 147 446–455. 10.1104/pp.108.118828 18524876 PMC2409020

[B28] Castle L. A. Siehl D. L. Gorton R. Patten P. A. Chen Y. H. Bertain S. (2004). Discovery and directed evolution of a glyphosate tolerance gene. *Science* 304 1151–1114. 10.1126/science.1096770 15155947

[B29] Cattivelli L. Rizza F. Badeck F. W. Mazzucottelli E. Mastrangelo A. M. Francia E. (2008). Drought tolerance improvement in crop plants: an integrated view from breeding to genomics. *Field Crop Res.* 105 1–14. 10.1016/j.fcr.2007.07.004

[B30] Cegielska-Taras T. Pniewski T. Szała L. (2008). Transformation of microspore-derived embryos of winter oilseed rape (*Brassica napus* L.) by using *Agrobacterium tumefaciens*. *J. Appl. Genet.* 49 343–347. 10.1007/BF03195632 19029681

[B31] Century K. Reuber T. L. Ratcliffe O. J. (2008). Regulating the regulators: the future prospects for transcription-factor based agricultural biotechnology products. *Plant Physiol.* 147 20–29. 10.1104/pp.108.117887 18443103 PMC2330319

[B32] Chapman K. Feussner I. (2016). Plant lipid biology. *Biochim. Biophys. Acta* 1861 1205–1206. 10.1016/j.bbalip.2016.05.005 27180218

[B33] Chen Y. Li Y. Zhou M. Cai Z. Tambel L. I. M. Zhang X. (2019). Nitrogen deficit decreases seed Cry1Ac endotoxin expression in Bt transgenic cotton. *Plant Physiol. Biochem.* 141 114–121. 10.1016/j.plaphy.2019.05.017 31146093

[B34] Chen Z. Gu H. Li Y. Su Y. Wu P. Jiang Z. (2003). Safety assessment for genetically modified sweet pepper and tomato. *Toxicology* 188 297–307. 10.1016/s0300-483x(03)00111-2 12767699

[B35] Chhapekar S. Raghavendrarao S. Pavan G. Ramakrishna C. Singh V. K. Phanindra M. L. V. (2015). Transgenic rice expressing a codon-modified synthetic CP4-EPSPS confers tolerance to broad-spectrum herbicide, glyphosate. *Plant Cell Rep.* 34 721–731. 10.1007/s00299-014-1732-2 25537885

[B36] Chiozza M. V. Burachik M. Miranda P. V. (2020). Compositional analysis of soybean event IND-ØØ41Ø-5. *GM Crop Food* 11 154–163. 10.1080/21645698.2020.1742040 32351157 PMC7518735

[B37] Chitchumroonchokchai C. Diretto G. Parisi B. Giuliano G. Failla M. L. (2017). Potential of golden potatoes to improve vitamin A and vitamin E status in developing countries. *PLoS One* 12:e0187102. 10.1371/journal.pone.0187102 29117188 PMC5678870

[B38] Cohen I. Zandalinas S. I Huck C. Fritschi F. B. Mittler R. (2021). Meta-analysis of drought and heat stress combination impact on crop yield and yield components. *Physiol. Plant* 171 66–76. 10.1111/ppl.13203 32880977

[B39] Cui J. Luo J. Van Der Werf W. Ma Y. Xia J. (2011). Effect of pyramiding Bt and CpTI genes on resistance of cotton to *Helicoverpa armigera* (Lepidoptera: Noctuidae) under laboratory and field conditions. *J. Econ. Entomol.* 104 673–684. 10.1603/ec09228 21510221

[B40] Cutler S. R. Rodriguez P. L. Finkelstein R. R. Abrams S. R. (2010). Abscisic acid: emergence of a core signaling network. *Ann. Rev. Plant Biol.* 61 651–679. 10.1146/annurev-arplant-042809-112122 20192755

[B41] Datta K. Baisakh N. Ganguly M. Krishnan S. Shinozaki K. Y. Datta S. K. (2012). Overexpression of *Arabidopsis* and rice stress genes inducible transcription factor confers drought and salinity tolerance to rice. *Plant Biotechnol. J.* 10 579–586. 10.1111/j.1467-7652.2012.00688.x 22385556

[B42] Davis M. J. Ying Z. (2004). Development of papaya breeding lines with transgenic resistance to papaya ringspot virus. *Plant Dis.* 88 352–358. 10.1094/PDIS.2004.88.4.352 30812613

[B43] Demaneche S. Sanguin H. Pote J. Navarro E. Bernillon D. Mavingui P. (2008). Antibiotic-resistant soil bacteria in transgenic plant fields. *Proc. Natl. Acad. Sci. U.S.A.* 105 3957–3962. 10.1073/pnas.0800072105 18292221 PMC2268783

[B44] Devos Y. Reheul D. de Schrijver A. Cors F. Moens W. (2004). Management of herbicide-tolerant oilseed rape in Europe: a case study on minimizing vertical gene flow. *Environ. Biosaf. Res.* 3 135–148. 10.1051/ebr:2005001 15901096

[B45] Dong T. Park Y. Hwang I. (2015). Abscisic acid: biosynthesis, inactivation, homoeostasis and signalling. *Essays Biochem.* 58 29–48. 10.1042/bse0580029 26374885

[B46] Dormann P. (2021). Plant lipid databases. *Method Mol. Biol.* 2295 441–454. 10.1007/978-1-0716-1362-7_25 34047992

[B47] Douglas A. E. (2018). Strategies for enhanced crop resistance to insect pests. *Annu. Rev. Plant Biol.* 69 637–660. 10.1146/annurev-arplant-042817-040248 29144774

[B48] Downes S. Kriticos D. Parry H. Paull C. Schellhorn N. Zalucki M. P. (2017). A perspective on management of *Helicoverpa armigera*: transgenic Bt cotton, IPM, and landscapes. *Pest Manag. Sci.* 73 485–492. 10.1002/ps.4461 27753247

[B49] Eckardt N. A. Cominelli E. Galbiati M. Tonelli C. (2009). The future of science: food and water for life. *Plant Cell* 21 368–372. 10.1105/tpc.109.066209 19252079 PMC2660623

[B50] EFSA Panel on Genetically Modified Organisms [GMO] Naegeli H. Bresson J. L. Dalmay T. Dewhurst I. C. Epstein M. M. (2018). Assessment of genetically modified maize MZHG0JG for food and feed uses, import and processing under regulation (EC) No 1829/2003 (application EFSA-GMO-DE-2016-133). *EFSA J.* 16:e05469. 10.2903/j.efsa.2018.5469 32625751 PMC7009398

[B51] EFSA Panel on Genetically Modified Organisms [GMO] Naegeli H. Bresson J. L. Dalmay T. Dewhurst I. C. Epstein M. M. (2020a). Assessment of genetically modified maize MZIR098 for food and feed uses, under regulation (EC) No 1829/2003 (application EFSA-GMO-DE-2017-142). *EFSA J.* 18:e06171. 10.2903/j.efsa.2020.6171 32874344 PMC7448020

[B52] EFSA Panel on Genetically Modified Organisms [GMO] Naegeli H. Bresson J. L. Dalmay T. Dewhurst I. C. Epstein M. M. (2020b). Assessment of genetically modified soybean SYHT0H2 for food and feed uses, import and processing, under regulation (EC) No 1829/2003 (application EFSA-GMO-DE-2012-111). *EFSA J.* 18:e05946. 10.2903/j.efsa.2020.5946 32626498 PMC7008876

[B53] Fang Y. Tyler B. M. (2016). Efficient disruption and replacement of an effector gene in the oomycete Phytophthora sojae using CRISPR/Cas9. *Mol. Plant Pathol.* 17 127–139. 10.1111/mpp.12318 26507366 PMC6638440

[B54] Farre G. Ramessar K. Twyman R. M. Capell T. Christou P. (2010). The humanitarian impact of plant biotechnology: recent breakthroughs vs bottlenecks for adoption. *Curr. Opin. Plant Biol.* 13 219–225. 10.1016/j.pbi.2009.11.002 20022290

[B55] Faure J. D. Napier J. A. (2018). Europe’s first and last field trial of gene-edited plants? *eLife* 7:e42379. 10.7554/eLife.42379 30558714 PMC6298765

[B56] Fiehn O. Weckwerth W. (2003). Deciphering metabolic networks. *Eur. J. Biochem.* 270 579–588. 10.1046/j.1432-1033.2003.03427.x 12581198

[B57] Foster S. J. Park T. H. Pel M. Brigneti G. Jones J. (2009). Rpi-vnt1.1, a tm-2(2) homolog from *Solanum venturii*, confers resistance to potato late blight. *Mol. Plant Microbe Interact.* 22 589–600. 10.1094/MPMI-22-5-0589 19348576

[B58] Fraley R. T. Rogers S. G. Horsc H. R. B. Sanders P. R. Flick J. S. Adams S. P. (1983). Expression of bacterial genes in plant cells. *Proc. Nat. Acad. Sci. U.S.A.* 80 4803–4807. 10.1073/pnas.80.15.4803 6308651 PMC384133

[B59] Fujii H. Chinnusamy V. Rodrigues A. Rubio S. Antoni R. Park S. Y. (2009). In vitro reconstitution of an abscisic acid signalling pathway. *Nature* 462 660–664. 10.1038/nature08599 19924127 PMC2803041

[B60] Funke T. Han H. Healy-Fried M. L. Fischer M. Schonbrunn E. (2006). Molecular basis for the herbicide resistance of roundup ready crops. *Proc. Nat. Acad. Sci. U.S.A.* 103 13010–13015. 10.1073/pnas.0603638103 16916934 PMC1559744

[B61] Gaj T. Gersbach C. A. Barbas C. F. III (2013). ZFN, TALEN, and CRISPR/Cas-based methods for genome engineering. *Trends Biotech.* 31 397–405. 10.1016/j.tibtech.2013.04.004 23664777 PMC3694601

[B62] Gao C. (2021). Genome engineering for crop improvement and future agriculture. *Cell* 184 1621–1635. 10.1016/j.cell.2021.01.005 33581057

[B63] Gao H. Wang Y. Xu P. Zhang Z. (2018). Overexpression of a WRKY transcription factor TaWRKY2 enhances drought stress tolerance in transgenic wheat. *Front. Plant Sci.* 9:997. 10.3389/fpls.2018.00997 30131813 PMC6090177

[B64] Gatehouse A. M. Ferry N. Edwards M. G. Bell H. A. (2011). Insect-resistant biotech crops and their impacts on beneficial arthropods. *Philos. Trans. R. Soc. Lond. B Biol. Sci.* 366 1438–1452. 10.1098/rstb.2010.0330 21444317 PMC3081576

[B65] Georges F. Ray H. (2017). Genome editing of crops: a renewed opportunity for food security. *GM Crop Food* 8 1–12. 10.1080/21645698.2016.1270489 28075688 PMC5592977

[B66] Ghareyazie B. Alinia F. Menguito C. A. Rubia L. G. de Palma J. M. Liwanag E. A. (1997). Enhanced resistance to two stem borers in an aromatic rice containing a synthetic cryIA(b) gene. *Mol. Breed.* 3 401–414. 10.1023/A:1009695324100

[B67] Gimenez E. Salinas M. Manzano-Agugliaro F. (2018). Worldwide research on plant defense against biotic stresses as improvement for sustainable agriculture. *Sustainability* 10:391. 10.3390/su10020391

[B68] Giraldo P. A. Shinozuka H. Spangenberg G. C. Cogan N. O. I. Smith K. F. (2019). Safety assessment of genetically modified feed: is there any difference from food? *Front. Plant Sci.* 10:1592. 10.3389/fpls.2019.01592 31921242 PMC6918800

[B69] Giudice G. Moffa L. Varotto S. Cardone M. F. Bergamini C. De Lorenzis G. (2021). Novel and emerging biotechnological crop protection approaches. *Plant Biotechnol. J.* 19 1495–1510. 10.1111/pbi.13605 33945200 PMC8384607

[B70] Guo B. Guo Y. Hong H. Jin L. Zhang L. Chang R. Z. (2015). Co-expression of G2-EPSPS and glyphosate acetyltransferase GAT genes conferring high tolerance to glyphosate in soybean. *Front. Plant Sci.* 6:847. 10.3389/fpls.2015.00847 26528311 PMC4606067

[B71] Halterman D. A. Kramer L. C. Wielgus S. Jiang J. (2008). Performance of transgenic potato containing the late blight resistance gene RB. *Plant Dis.* 92 339–343. 10.1094/PDIS-92-3-0339 30769693

[B72] Hartung F. Schiemann J. (2014). Precise plant breeding using new genome editing techniques: opportunities, safety and regulation in the EU. *Plant J.* 78 742–752. 10.1111/tpj.12413 24330272

[B73] Haslam R. P. Sayanova O. Kim H. J. Cahoon E. B. Napier J. A. (2016). Synthetic redesign of plant lipid metabolism. *Plant J.* 87 76–86. 10.1111/tpj.13172 27483205 PMC4982047

[B74] Herman R. A. Fast B. J. Johnson T. Y. Sabbatini J. Rudgers G. W. (2013). Compositional safety of herbicide-tolerant DAS-81910-7 cotton. *J. Agric. Food Chem.* 61 11683–11692. 10.1021/jf404043y 24147981

[B75] Hernández-Soto A. Chacón-Cerdas R. (2021). RNAi crop protection advances. *Int. J. Mol. Sci.* 22:12148. 10.3390/ijms222212148 34830030 PMC8625170

[B76] Hérouet C. Esdaile D. J. Mallyon B. A. Debruyne E. Schulz A. Currier T. (2005). Safety evaluation of the phosphinothricin acetyltransferase proteins encoded by the pat and bar sequences that confer tolerance to glufosinate-ammonium herbicide in transgenic plants. *Regul. Toxicol. Pharmacol.* 41 134–149. 10.1016/j.yrtph.2004.11.002 15698537

[B77] Herrera-Estrella L. Depicker A. Van Montagu M. Schell J. (1983). Expression of chimaeric genes transferred into plant cells using a Ti-plasmid-derived vector. *Nature* 303 209–213. 10.1038/303209a0 1422044

[B78] Hu J. J. Tian Y. C. Han Y. F. Li Y. Zhang B. E. (2001). Field evaluation of insect-resistant transgenic *Populus nigra* trees. *Euphytica* 121 123–127.

[B79] Hu X. Liu L. Xiao B. Li D. Xing X. Kong X. (2010). Enhanced tolerance to low temperature in tobacco by over-expression of a new maize protein phosphatase 2C, ZmPP2C2. *J. Plant Physiol.* 167 1307–1315. 10.1016/j.jplph.2010.04.014 20580122

[B80] Hu X. Wang C. Fu Y. Liu Q. Jiao X. Wang K. (2016). Expanding the range of CRISPR/Cas9 genome editing in rice. *Mol. Plant* 9 943–945. 10.1016/j.molp.2016.03.003 26995294

[B81] Huang J. Sun S. J. Xu D. Q. Yang X. Bao Y. M. Wang Z. F. (2009). Increased tolerance of rice to cold, drought and oxidative stresses mediated by the overexpression of a gene that encodes the zinc finger protein ZFP245. *Biochem. Biophys. Res. Commun.* 389 556–561. 10.1016/j.bbrc.2009.09.032 19751706

[B82] Hüsken A. Dietz-Pfeilstetter A. (2007). Pollen-mediated intraspecific gene flow from herbicide resistant oilseed rape (*Brassica napus* L.). *Transgenic Res.* 16 557–569. 10.1007/s11248-007-9078-y 17541721

[B83] Hussain A. Ding X. Alariqi M. Manghwar H. Hui F. Li Y. (2021). Herbicide resistance: another hot agronomic trait for plant genome editing. *Plants* 10:621. 10.3390/plants10040621 33805182 PMC8064318

[B84] Ilardi V. Nicola-Negri E. D. (2011). Genetically engineered resistance to plum pox virus infection in herbaceous and stone fruit hosts. *GM Crop* 2 24–33. 10.4161/gmcr.2.1.15096 21844696

[B85] ISAAA (2022). *GM Approval Database.* Available online at: http://www.isaaa.org/gmapprovaldatabase/default.asp (accessed June 11, 2022).

[B86] Ishino Y. Shinagawa H. Makino K. Amemura M. Nakata A. (1987). Nucleotide sequence of the IAP gene, responsible for alkaline phosphatase isozyme conversion in *Escherichia coli*, and identification of the gene product. *J. Bacteriol.* 169 5429–5433. 10.1128/jb.169.12.5429-5433.1987 3316184 PMC213968

[B87] Jacobsen E. Schouten H. J. (2009). Cisgenesis: an important sub-invention for traditional plant breeding companies. *Euphytica* 170 235–247. 10.1007/s10681-009-0037-y

[B88] Jaganathan D. Ramasamy K. Sellamuthu G. Jayabalan S. Venkataraman G. (2018). CRISPR for crop improvement: an update review. *Front. Plant Sci.* 9:985. 10.3389/fpls.2018.00985 30065734 PMC6056666

[B89] Kacser H. Burns J. A. (1995). The control of flux. *Biochem. Soc. Trans.* 23 341–366. 7672373 10.1042/bst0230341

[B90] Kamthan A. Chaudhuri A. Kamthan M. Datta A. (2016). Genetically modified (GM) crops: milestones and new advances in crop improvement. *Theor. Appl. Genet.* 129 1639–1655. 10.1007/s00122-016-2747-6 27381849

[B91] Karthik K. Nandiganti M. Thangaraj A. Singh S. Mishra P. Rathinam M. (2020). Transgenic cotton (*Gossypium hirsutum* L.) to combat weed vagaries: utility of an apical meristem-targeted in planta transformation strategy to introgress a modified CP4-EPSPS gene for glyphosate tolerance. *Front. Plant Sci.* 11:768. 10.3389/fpls.2020.00768 32733492 PMC7358616

[B92] Kasuga M. Liu Q. Miura S. Yamaguchi-Shinozaki Y. Shinozaki K. (1999). Improving plant drought, salt, and freezing tolerance by gene transfer of a single stress-inducible transcription factor. *Nat. Biotechnol.* 17 287–291. 10.1038/7036 10096298

[B93] Katta S. Talakayala A. Reddy M. K. Addepally U. Garladinne M. (2020). Development of transgenic cotton (Narasimha) using triple gene cry2Ab-cry1F-cry1Ac construct conferring resistance to lepidopteran pests. *J. Biosci.* 45:31. 32020913

[B94] Khan S. Anwar S. Yu S. Sun M. Yang Z. Gao Z. (2019). Development of drought-tolerant transgenic wheat: achievements and limitations. *Int. J. Mol. Sci.* 20:3350. 10.3390/ijms20133350 31288392 PMC6651533

[B95] Kim D. Alptekin B. Budak H. (2018). CRISPR/Cas9 genome editing in wheat. *Funct. Integr. Genomics* 18 31–41. 10.1007/s10142-017-0572-x 28918562

[B96] King A. J. Montes L. R. Clarke J. G. Itzep J. Perez C. A. A. Jongschaap R. E. E. (2015). Identification of QTL markers contributing to plant growth, oil yield and fatty acid composition in the oilseed crop *Jatropha curcas* L. *Biotechnol. Biofuels* 8:160. 10.1186/s13068-015-0326-8 26413159 PMC4583170

[B97] Kinney A. J. (1996). Development of genetically engineered soybean oils for food applications. *J. Food Lipid.* 3 273–292. 10.1111/j.1745-4522.1996.tb00074.x

[B98] Kinney A. J. Cahoon E. B. Hitz W. D. (2022). Manipulating desaturase activities in transgenic crop plants. *Biochem. Soc. Trans.* 30, 1099–1103. 10.1042/bst0301099 12440982

[B99] Klee H. J. (1993). Ripening physiology of fruit from transgenic tomato (*Lycopersicon esculentum*) plants with reduced ethylene synthesis. *Plant Physiol.* 102 911–916. 10.1104/pp.102.3.911 12231876 PMC158863

[B100] Klumper W. Qaim M. (2014). A meta-analysis of the impacts of genetically modified crops. *PLoS One* 9:e111629. 10.1371/journal.pone.0111629 25365303 PMC4218791

[B101] Knutzon D. S. Thurmond J. M. Huang Y. S. Chaudhary S. Bobik E. G. Jr. Chan G. M. (1998). Identification of delta 5-desaturase from *Mortierella alpina* by heterologous expression in Bakers’ yeast and canola. *J. Biol. Chem.* 273 29360–29366. 10.1074/jbc.273.45.29360 9792636

[B102] Koc I. Filiz E. Tombuloglu H. (2015). Comparative analysis of plant lycopene cyclases. *Comput. Biol. Chem.* 58 81–92. 10.1016/j.compbiolchem.2015.06.001 26092704

[B103] Kovalchuk N. Chew W. Sornaraj P. Borisjuk N. Yang N. Singh R. (2016). The homeodomain transcription factor TaHDZipI-2 from wheat regulates frost tolerance, flowering time and spike development in transgenic barley. *New Phytol.* 211 671–687. 10.1111/nph.13919 26990681

[B104] Langner T. Kamoun S. Belhaj K. (2018). CRISPR crops: plant genome editing toward disease resistance. *Annu. Rev. Phytopathol.* 56 479–512. 10.1146/annurev-phyto-080417-050158 29975607

[B105] Lawrenson T. Shorinola O. Stacey N. Li C. Østergaard L. Patron N. (2015). Induction of targeted, heritable mutations in barley and *Brassica oleracea* using RNA-guided Cas9 nuclease. *Genome Biol.* 16:258. 10.1186/s13059-015-0826-7 26616834 PMC4663725

[B106] Légère A. (2005). Risks and consequences of gene flow from herbicide-resistant crops: canola (*Brassica napus* L) as a case study. *Pest Manag. Sci.* 61 292–300. 10.1002/ps.975 15593291

[B107] Leibowitz M. L. Papathanasiou S. Doerfler P. A. Blaine L. J. Sun L. Yao Y. (2021). Chromothripsis as an on-target consequence of CRISPR–Cas9 genome editing. *Nat. Genet.* 53 889–905. 10.1038/s41588-021-00838-7 33846636 PMC8192433

[B108] Li J. Jiao G. Sun Y. Chen J. Zhong Y. Yan L. (2020). Modification of starch composition, structure and properties through editing of TaSBEIIa in both winter and spring wheat varieties by CRISPR/Cas9. *Plant Biotech.* 19 937–951. 10.1111/pbi.13519 33236499 PMC8131058

[B109] Li J. Zhang H. Si X. Tian Y. Chen K. Liu J. (2017). Generation of thermosensitive male-sterile maize by targeted knockout of the ZmTMS5 gene. *J. Genet. Genomics* 44 465–468. 10.1016/j.jgg.2017.02.002 28412227

[B110] Li M. Li X. Zhou Z. Wu P. Fang M. Pan X. (2016). Reassessment of the four yield-related genes Gn1a, DEP1, GS3, and IPA1 in rice using a CRISPR/Cas9 system. *Front. Plant Sci.* 7:377. 10.3389/fpls.2016.00377 27066031 PMC4811884

[B111] Li S. Zhao Q. Zhu D. Yu J. (2018). A DREB-like transcription factor from maize (*Zea mays*), ZmDREB4.1, plays a negative role in plant growth and development. *Front. Plant Sci.* 9:395. 10.3389/fpls.2018.00395 29670637 PMC5893645

[B112] Liang C. Z. Sun B. Meng Z. G. Meng Z. H. Wang Y. Sun G. Q. (2017). Co-expression GR79 EPSPS and GAT yields herbicide-resistant cotton with low glyphosate residues. *Plant Biotechnol. J.* 15 1622–1629. 10.1111/pbi.12744 28418615 PMC5698046

[B232] Liang Z. Zhang K. Chen K. Gao C. (2014). Targeted mutagenesis in *Zea mays* using TALENs and the CRISPR/Cas system. *J. Genet. Genomics*. 41, 63–68. 10.1016/j.jgg.2013.12.001 24576457

[B113] Liu D. (2009). Design of gene constructs for transgenic maize. *Method Mol. Biol.* 526 3–20. 10.1007/978-1-59745-494-0_1 PMC7121396 19378011

[B114] Ma Y. Szostkiewicz I. Korte A. Moes D. Yang Y. Christmann A. (2009). Regulators of PP2C phosphatase activity function as abscisic acid sensors. *Science* 324 1064–1068. 10.1126/science.1172408 19407143

[B115] Malnoy M. Viola R. Jung M. H. Koo O. J. Kim S. Kim J. S. (2016). DNA-free genetically edited grapevine and apple protoplast using CRISPR/Cas9 ribonucleoproteins. *Front. Plant Sci.* 7:1904. 10.3389/fpls.2016.01904 28066464 PMC5170842

[B116] Mamta B. Rajam M. V. (2017). RNAi technology: a new platform for crop pest control. *Physiol. Mol. Biol. Plants* 23 487–501. 10.1007/s12298-017-0443-x 28878489 PMC5567704

[B117] Matten S. R. Reynolds A. H. (2003). Current resistance management requirements for Bt cotton in the United States. *J. New Seeds* 5 137–178. 10.1300/J153v05n02_04

[B118] McNaughton J. Roberts M. Smith B. Rice D. Hinds M. Rood T. (2008). Comparison of broiler performance and carcass yields when fed diets containing transgenic maize grains from event DP-O9814O-6 (Optimum GAT), near-isogenic control maize grain, or commercial reference maize grains. *Poult Sci.* 87 2562–2572. 10.3382/ps.2008-00017 19038812

[B119] Melo A. L. Soccol V. T. Soccol C. R. (2016). *Bacillus thuringiensis*: mechanism of action, resistance, and new applications: a review. *Crit. Rev. Biotechnol.* 36 317–326. 10.3109/07388551.2014.960793 25264571

[B120] Molla K. A. Karmakar S. Islam M. T. (2020). “Wide horizons of CRISPR-Cas-derived technologies for basic biology, agriculture, and medicine,” in *CRISPR-Cas Methods. Springer Protocols Handbooks*, eds Islam M. T. Bhowmik P. K. Molla K. A. (New York, NY: Humana).

[B121] Molla K. A. Sretenovic S. Bansal K. C. Qi Y. (2021). Precise plant genome editing using base editors and prime editors. *Nat. Plants* 7 1166–1187. 10.1038/s41477-021-00991-1 34518669

[B122] Molla K. A. Yang Y. (2019). CRISPR/Cas-mediated base editing: technical considerations and practical applications. *Trends Biotech.* 37 1121–1142. 10.1016/j.tibtech.2019.03.008 30995964

[B123] Monsur M. B. Cao N. Wei X. Xie L. Jiao G. Tang S. (2021). Improved eating and cooking quality of indica rice cultivar YK17 via adenine base editing of waxy A allele of granule-bound starch synthase I (GBSS I). *Rice Sci.* 12 427–430. 10.1016/j.rsci.2021.07.003

[B124] Murai N. Kemp J. D. Sutton D. W. Murray M. G. Slightom J. L. Merlo D. J. (1983). Phaseolin gene from bean is expressed after transfer to sunflower via tumor-inducing plasmid vectors. *Science* 222 476–482. 10.1126/science.222.4623.476 17746179

[B125] Naeem M. Majeed S. Hoque M. Z. Ahmad I. J. C. (2020). Latest developed strategies to minimize the off-target effects in CRISPR-Cas mediated genome editing. *Cells* 9:1608. 10.3390/cells9071608 32630835 PMC7407193

[B126] Napier J. A. Haslam R. P. Tsalavouta M. Sayanova O. (2019). The challenges of delivering genetically modified crops with nutritional enhancement traits. *Nat. Plants* 5 563–567. 10.1038/s41477-019-0430-z 31160704

[B127] Naranjo S. E. (2011). Impacts of Bt transgenic cotton on integrated pest management. *J. Agric. Food Chem.* 59 5842–5851. 10.1021/jf102939c 20942488

[B128] Nekrasov V. Staskawicz B. Weigel D. Jones D. Kamoun S. (2013). Targeted mutagenesis in the model plant *Nicotiana enthamiana* using Cas9 RNA-guided endonuclease. *Nat. Biotech.* 31 691–693. 10.1038/nbt.2655 23929340

[B129] Nelson D. D. Repetti P. P. Adams T. T. Creelman R. R. Wu J. Warner D. D. (2007). Plant nuclear factor Y (NF-Y) B subunits confer drought tolerance and lead to improved corn yields on water-limited acres. *Proc. Natl. Acad. Sci. U.S.A.* 104 16450–16455. 10.1073/pnas.0707193104 17923671 PMC2034233

[B130] Oerke E. C. (2006). Crop losses to pests. *J. Agric. Sci.* 144 31–43. 10.1017/S0021859605005708

[B131] Okuzaki A. Ogawa T. Koizuka C. Kaneko K. Inaba M. Imamura J. (2018). CRISPR/Cas9-mediated genome editing of the fatty acid desaturase 2 gene in Brassica napus. *Plant Physiol. Biochem.* 131 63–69. 10.1016/j.plaphy.2018.04.025 29753601

[B132] Orbegozo J. Solorzano D. Cuellar W. J. Bartolini I. Roman M. L. Ghislain M. (2016). Marker-free PLRV resistant potato mediated by Cre-loxP excision and RNAi. *Transgenic Res.* 25 813–828. 10.1007/s11248-016-9976-y 27544267 PMC5104775

[B133] Ortiz R. Iwanaga M. Reynolds M. P. Wu H. Crouch J. H. (2007). Overview on crop genetic engineering for drought-prone environments. *J. Sat Agri. Res.* 4:30. 10.1093/jxb/erh269 15475377

[B134] Otani M. Hamada T. Katayama K. Kitahara K. Kim S. H. Takahata Y. (2007). Inhibition of the gene expression for granule-bound starch synthase I by RNA interference in sweet potato plants. *Plant Cell Rep.* 26 1801–1807. 10.1007/s00299-007-0396-6 17622537

[B135] Padgette S. R. Kolacz K. H. Delannay X. Re D. B. Lavallee B. G. Tinius C. N. (1995). Development, identification, and characterization of a glyphosate-tolerant soybean line. *Crop Sci.* 35 1451–1461. 10.2135/cropsci1995.0011183X003500050032x

[B136] Paine J. A. Shipton C. A. Chaggar S. Howells R. M. Kennedy M. J. Vernon G. (2005). Improving the nutritional value of Golden Rice through increased pro-vitamin A content. *Nat. Biotechnol.* 23 482–487. 10.1038/nbt1082 15793573

[B137] Palma L. Muñoz D. Berry C. Murillo J. Caballero P. (2014). *Bacillus thuringiensis* toxins: an overview of their biocidal activity. *Toxins* 6 3296–3325. 10.3390/toxins6123296 25514092 PMC4280536

[B138] Panthi S. Sapkota A. R. Raspanti G. Allard S. M. Bui A. Craddock H. A. (2019). Pharmaceuticals, herbicides, and disinfectants in agricultural water sources. *Environ. Res.* 174 1–8. 10.1016/j.envres.2019.04.011 31015109

[B139] Park S. Y. Fung P. Nishimura N. Jensen D. R. Fujii H. Zhao Y. (2009). Abscisic acid inhibits type 2C protein phosphatases via the PYR/PYL family of START proteins. *Science* 324 1068–1071. 10.1126/science.1173041 19407142 PMC2827199

[B140] Parrott W. Chassy B. Ligon J. Meyer L. Petrick J. Zhou J. (2010). Application of food and feed safety assessment principles to evaluate transgenic approaches to gene modulation in crops. *Food Chem. Toxicol.* 48 1773–1790. 10.1016/j.fct.2010.04.017 20399824

[B141] Passioura J. B. (2012). Phenotyping for drought tolerance in grain crops: when is it useful to breeders? *Funct. Plant Biol.* 39 851–859. 10.1071/FP12079 32480835

[B142] Pierre C. C. Crossa J. J. Bonnett D. Yamaguchi-Shinozaki K. Reynolds M. P. (2012). Phenotyping transgenic wheat for drought resistance. *J. Exp. Bot.* 63 1799–1808. 10.1093/jxb/err385 22213810

[B143] Poongothai S. Iiavarasan R. Karrunakaran C. M. (2010). Cry1Ac levels and biochemical variations in Bt cotton as influenced by tissue maturity and senescence. *J. Plant Breed. Crop Sci.* 2 96–103.

[B144] Pray C. Ma D. Huang J. Qiao F. (2001). Impact of Bt cotton in China. *World Dev.* 29 813–825. 10.1016/S0305-750X(01)00010-9

[B145] Preuss S. B. Meister R. Xu Q. Urwin C. P. Tripodi F. A. Screen S. E. (2012). Expression of the *Arabidopsis thaliana* BBX32 gene in soybean increases grain yield. *PLoS One* 7:e30717. 10.1371/journal.pone.0030717 22363475 PMC3281879

[B146] Ramachandra D. Ramamohan G. Bhan A. Suresh P. J. (2016). Weed management in cotton: the potential of GM crops. *Indian J. Weed Sci.* 48 136–143. 10.5958/0974-8164.2016.00035.6

[B147] Ramaseshadri P. Segers G. Flannagan R. Wiggins E. Clinton W. Ilagan O. (2013). Physiological and cellular responses caused by RNAi- mediated suppression of Snf7 orthologue in western corn rootworm (*Diabrotica virgifera virgifera*) larvae. *PLoS One* 8:e54270. 10.1371/journal.pone.0054270 23349844 PMC3548817

[B148] Rasheed A. Gill R. A. Hassan M. U. Mahmood A. Qari S. Zaman Q. U. (2021). Critical review: recent advancements in the use of CRISPR/Cas9 technology to enhance crops and alleviate global food crises. *Curr. Issues Mol. Biol.* 43 1950–1976. 10.3390/cimb43030135 34889892 PMC8929161

[B149] Raymond Park J. Mcfarlane I. Hartley, Phipps R. Ceddia G. (2011). The role of transgenic crops in sustainable development. *Plant Biotechnol. J.* 9 2–21. 10.1111/j.1467-7652.2010.00565.x 21040386

[B150] Ribichich K. F. Chiozza M. Avalos-Britez S. Cabello J. V. Arce A. L. Watson G. (2020). Successful field performance in warm and dry environments of soybean expressing the sunflower transcription factor HaAb4. *J. Exp. Bot.* 71 3142–3156. 10.1093/jxb/eraa064 32140724 PMC7260725

[B151] Rice E. A. Khandelwal A. Creelman R. A. Griffith C. Ahrens J. E. Taylor J. P. (2014). Expression of a truncated ATHB17 protein in maize increases ear weight at silking. *PLoS One* 9:e94238. 10.1371/journal.pone.0094238 24736658 PMC3988052

[B152] Rocha-Munive M. G. Soberon M. Castaneda S. Niaves E. Scheinvar E. Eguiarte L. E. (2018). Evaluation of the impact of genetically modified cotton after 20 years of cultivation in Mexico. *Front. Bioeng. Biotechnol.* 6:82. 10.3389/fbioe.2018.00082 29988354 PMC6023983

[B153] Rommens C. M. Haring M. A. Swords K. Davies H. V. Belknap W. R. (2007). The intragenic approach as a new extension of traditional plant breeding. *Trends Plant Sci.* 12 397–403. 10.1016/j.tplants.2007.08.001 17692557

[B154] Samaras A. Karaoglanidis G. S. Tzelepis G. (2021). Insights into the multitrophic interactions between the biocontrol agent *Bacillus subtilis* MBI 600, the pathogen Botrytis cinerea and their plant host. *Microbiol. Res.* 248:126752. 10.1016/j.micres.2021.126752 33839506

[B155] Sanahuja G. Banakar R. Twyman R. M. Capell T. Christou P. (2011). *Bacillus thuringiensis*: a century of research, development and commercial applications. *Plant Biotechnol. J.* 9 283–300. 10.1111/j.1467-7652.2011.00595.x 21375687

[B156] Sarkar T. Thankappan R. Mishra G. P. Nawade B. D. (2019). Advances in the development and use of DREB for improved abiotic stress tolerance in transgenic crop plants. *Physiol. Mol. Biol. Plants* 25 1323–1334. 10.1007/s12298-019-00711-2 31736537 PMC6825097

[B157] Schilling R. K. Tester M. Marschner P. Plett D. C. Roy S. J. (2017). AVP1: one protein, many roles. *Trends Plant Sci.* 22 154–162. 10.1016/j.tplants.2016.11.012 27989652

[B158] Schouten H. J. Jacobsen E. (2008). Cisgenesis and intragenesis, sisters in innovative plant breeding. *Trends Plant Sci.* 13 260–261. 10.1016/j.tplants.2008.04.005 18486525

[B159] Schouten H. J. Krens F. A. Jacobsen E. (2006). Do cisgenic plants warrant less stringent oversight? *Nat. Biotechnol.* 24:753. 10.1038/nbt0706-753 16841052

[B160] Schuler T. H. Poppy G. M. Kerry B. R. Denholm I. (1998). Insect-resistant transgenic plants. *Trends Biotechnol.* 16 168–175. 10.1016/S0167-7799(97)01171-2 10322447

[B161] Shan Q. Wang Y. Li J. Zhang Y. Chen K. Liang Z. (2013). Targeted genome modification of crop plants using a CRISPR/Cas system. *Nat. Biotechnol.* 31 686–688. 10.1038/nbt.2650 23929338

[B162] Sharkey S. M. Williams B. J. Parker K. M. (2021). Herbicide drift from genetically engineered herbicide-tolerant crops. *Environ. Sci. Technol.* 55 15559–15568. 10.1021/acs.est.1c01906 34813302

[B163] Sharma S. Vakhlu J. (2021). Evolution and biology of CRISPR system: a new era tool for genome editing in plants. *Bot. Rev.* 2021 1–22. 10.1007/s12229-021-09250-6

[B164] Shen L. Wang C. Fu Y. Wang J. Liu Q. Zhang X. (2018). QTL editing confers opposing yield performance in different rice varieties. *J. Integr. Plant Biol.* 60 89–93. 10.1111/jipb.12501 27628577

[B165] Shi J. Gao H. Wang H. Lafitte H. R. Archibald R. L. Yang M. (2017). ARGOS8 variants generated by CRISPR-Cas9 improve maize grain yield under field drought stress conditions. *Plant Biotechnol. J.* 15 207–216. 10.1111/pbi.12603 27442592 PMC5258859

[B166] Shimatani Z. Kashojiya S. Takayama M. Terada R. Arazoe T. Ishii H. (2017). Targeted base editing in rice and tomato using a CRISPR/Cas9 cytidine deaminase fusion. *Nat. Biotechnol.* 35 441–443. 10.1038/nbt.3833 28346401

[B167] Showalter A. M. Heuberger S. Tabashnik B. E. Carriere Y. Coates B. (2009). A primer for using transgenic insecticidal cotton in developing countries. *J. Insect Sci.* 9:22. 10.1673/031.009.2201 19613464 PMC3011844

[B168] Siehl D. L. Castle L. A. Gorton R. Keenan R. J. (2007). The molecular basis of glyphosate resistance by an optimized microbial acetyltransferase. *J. Biol. Chem.* 282 11446–11455. 10.1074/jbc.M610267200 17272278

[B169] Smyth S. Phillips P. W. B. Castle D. (2014). Benefits of genetically modified herbicide tolerant canola in Western Canada. *Int. J. Biotechnol.* 13:181. 10.1504/IJBT.2014.068928 35009967

[B170] Song J. Bradeen J. M. Naess S. K. Raasch J. A. Wielgus S. M. Haberlach G. T. (2003). Gene RB cloned from *Solanum bulbocastanum* confers broad spectrum resistance to potato late blight. *Proc. Natl. Acad. Sci. U.S.A.* 100 9128–9133. 10.1073/pnas.1533501100 12872003 PMC170883

[B171] Stalker D. M. Mcbride K. E. Malyj L. D. (1988). Herbicide resistance in transgenic plants expressing a bacterial detoxification gene. *Science* 242 419–423. 10.1126/science.242.4877.419 17789813

[B172] Takagi H. Hiroi T. Yang L. Tada Y. Yuki Y. Takamura K. (2005). A rice-based edible vaccine expressing multiple T cell epitopes induces oral tolerance for inhibition of Th2-mediated IgE responses. *Proc. Natl. Acad. Sci. U.S.A.* 102 17525–17530. 10.1073/pnas.0503428102 16278301 PMC1297655

[B173] Talakayala A. Katta S. Garladinne M. (2020). Genetic engineering of crops for insect resistance: an overview. *J. Biosci.* 45:114. 33051408

[B174] Tang X. Lowder L. G. Zhang T. Malzahn A. A. Zheng X. Voytas D. F. (2017). A CRISPR-Cpf1 system for efficient genome editing and transcriptional repression in plants. *Nat. Plants* 3:17103. 10.1038/nplants.2017.103 28628131

[B175] Taning C. N. T. Arpaia S. Christiaens O. Dietz-Pfeilstetter A. Jones H. Mezzetti B. (2020). RNA-based biocontrol compounds: current status and perspectives to reach the market. *Pest Manag. Sci.* 76 841–845. 10.1002/ps.5686 31743573

[B176] Tennant P. Souza M. T. Fitch M. M. Manshardt R. M. Slightom J. L. (2005). Line 63-1: a new virus-resistant transgenic papaya. *Hortscience* 40 1196–1199. 10.21273/HORTSCI.40.5.1196 35581909

[B177] Thelen J. J. Ohlrogge J. B. (2002). Metabolic engineering of fatty acid biosynthesis in plants. *Metab. Eng.* 4 12–21. 10.1006/mben.2001.0204 11800570

[B178] Thompson C. J. Movva N. R. Tizard R. Crameri R. Davies J. E. Lauwereys M. (1987). Characterization of the herbicide-resistance gene bar from *Streptomyces hygroscopicus*. *EMBO J.* 6 2519–2523. 10.1002/j.1460-2075 16453790 PMC553668

[B179] Thorup T. A. Tanyolac B. Livingstone K. D. Popovsky S. Paran I. Jahn M. (2000). Candidate gene analysis of organ pigmentation loci in the Solanaceae. *Proc. Natl. Acad. Sci. U.S.A.* 97 11192–11197. 10.1073/pnas.97.21.11192 11027328 PMC17176

[B180] Tollefson J. (2011). Drought-tolerant maize gets US debut. *Nature* 469:144. 10.1038/469144a 21228846

[B181] Tran L. S. Nishiyama R. Yamaguchi-Shinozaki K. Shinozaki K. (2010). Potential utilization of NAC transcription factors to enhance abiotic stress tolerance in plants by biotechnological approach. *GM Crops* 1 32–39. 10.4161/gmcr.1.1.10569 21912210

[B182] Turnbull C. Lillemo M. Hvoslef-Eide T. A. (2021). Global regulation of genetically modified crops amid the gene edited crop boom-a review. *Front. Plant Sci.* 12:630396. 10.3389/fpls.2021.630396 33719302 PMC7943453

[B183] Umezawa T. Sugiyama N. Takahashi F. Anderson J. C. Ishihama Y. Peck S. C. (2013). Genetics and phosphoproteomics reveal a protein phosphorylation network in the abscisic acid signaling pathway in *Arabidopsis thaliana*. *Sci. Signal.* 6:rs8. 10.1126/scisignal.2003509 23572148

[B184] Unger E. Cigan A. M. Trimnell M. Xu R. J. Kendall T. Roth B. (2002). A chimeric ecdysone receptor facilitates methoxyfenozide-dependent restoration of male fertility in ms45 maize. *Transgenic Res.* 11 455–465. 10.1023/a:1020350208095 12437077

[B185] Usman B. Nawaz G. Zhao N. Liao S. Qin B. Liu F. (2021). Programmed editing of rice (*Oryza sativa* L.) OsSPL16 gene using CRISPR/Cas9 improves grain yield by modulating the expression of pyruvate enzymes and cell cycle proteins. *Int. J. Mol. Sci.* 22:249. 10.3390/ijms22010249 33383688 PMC7795130

[B186] Usman B. Nawaz G. Zhao N. Liu Y. Li R. (2020). Generation of high yielding and fragrant rice (*Oryza sativa* L.) lines by CRISPR/Cas9 targeted mutagenesis of three homoeologs of cytochrome P450 gene family and OsBADH2 and transcriptome and proteome profiling of revealed changes triggered by mutations. *Plants* 9:788. 10.3390/plants9060788 32586052 PMC7355857

[B187] Vandenberg L. N. Blumberg B. Antoniou M. N. Benbrook C. M. Carroll L. Colborn T. (2017). Is it time to reassess current safety standards for glyphosate-based herbicides? *J. Epidemiol. Community Health* 71 613–618. 10.1136/jech-2016-208463 28320775 PMC5484035

[B188] Varshney R. K. Bansal K. C. Aggarwal P. K. Datta S. K. Craufurd P. Q. (2011). Agricultural biotechnology for crop improvement in a variable climate: hope or hype? *Trends Plant Sci.* 16 363–371. 10.1016/j.tplants.2011.03.004 21497543

[B189] Voelker T. Kinney A. J. (2001). Variations in the biosynthesis of seed-storage lipids. *Ann. Rev. Plant Physiol. Mol. Biol.* 52 335–361. 10.1146/annurev.arplant.52.1.335 11337402

[B190] Wally O. Punja Z. K. (2010). Genetic engineering for increasing fungal and bacterial disease resistance in crop plants. *GM Crops* 1 199–206. 10.4161/gmcr.1.4.13225 21844674

[B191] Waltz E. (2016a). Gene-edited CRISPR mushroom escapes US regulation. *Nature* 532:293. 10.1038/nature.2016.19754 27111611

[B192] Waltz E. (2016b). CRISPR-edited crops free to enter market, skip regulation. *Nat. Biotechnol.* 34:582. 10.1038/nbt0616-582 27281401

[B193] Wan H. Cui Y. Ding Y. Mei J. Dong H. Zhang W. (2017). Time-series analyses of transcriptomes and proteomes reveal molecular networks underlying oil accumulation in canola. *Front. Plant Sci.* 7:2007. 10.3389/fpls.2016.02007 28119706 PMC5222877

[B194] Wang P. Xue L. Batelli G. Lee S. Hou Y. J. Van Oosten M. J. (2013). Quantitative phosphoproteomics identifies SnRK2 protein kinase substrates and reveals the effectors of abscisic acid action. *Proc. Natl. Acad. Sci. U.S.A.* 110 11205–11210. 10.1073/pnas.1308974110 23776212 PMC3703982

[B195] Wang W. Wang X. Wang Y. Zhou G. Wang C. Hussain S. (2020). SlEAD1, an EAR motif-containing ABA down-regulated novel transcription repressor regulates ABA response in tomato. *GM Crops Food* 11 275–289. 10.1080/21645698.2020.1790287 32706315 PMC7518750

[B196] Wang Y. Cheng X. Shan Q. Zhang Y. Liu J. Gao C. (2014). Simultaneous editing of three homoeoalleles in hexaploid bread wheat confers heritable resistance to powdery mildew. *Nat. Biotechnol.* 32 947–951. 10.1038/nbt.2969 25038773

[B197] Wang Y. Wang J. Guo S. Tian S. Zhang J. Ren Y. (2021). CRISPR/Cas9-mediated mutagenesis of ClBG1 decreased seed size and promoted seed germination in watermelon. *Hortc. Res.* 8 1–12. 10.1038/s41438-021-00506-1 33790265 PMC8012358

[B198] Wang Y. G. Fu F. L. Yu H. Q. Hu T. Zhang Y. Y. Tao Y. (2018). Interaction network of core ABA signaling components in maize. *Plant Mol. Biol.* 96 245–263. 10.1007/s11103-017-0692-7 29344831

[B199] Wei W. Liang D. W. Bian X. H. Shen M. Xiao J. H. Zhang W. K. (2019). GmWRKY54 improves drought tolerance through activating genes in abscisic acid and Ca2+ signaling pathways in transgenic soybean. *Plant J.* 100 384–398. 10.1111/tpj.14449 31271689

[B200] Wolt J. D. Wang K. Yang B. (2016). The regulatory status of genome-edited crops. *Plant Biotechnol J.* 14 510–518. 10.1111/pbi.12444 26251102 PMC5042095

[B201] Wu X. Shiroto Y. Kishitani S. Ito Y. Toriyama K. (2009). Enhanced heat and drought tolerance in transgenic rice seedlings overexpressing OsWRKY11 under the control of HSP101 promoter. *Plant Cell Rep.* 28 21–30. 10.1007/s00299-008-0614-x 18818929

[B202] Wu Z. Mo C. Zhang S. Li H. (2018). Characterization of papaya ringspot virus isolates infecting transgenic papaya ‘Huanong No.1’ in South China. *Sci. Rep.* 8:8206. 10.1038/s41598-018-26596-x 29844514 PMC5974079

[B203] Xiang Y. Sun X. Gao S. Qin F. Dai M. (2017). Deletion of an endoplasmic reticulum stress response element in a ZmPP2C-A gene facilitates drought tolerance of maize seedlings. *Mol. Plant* 10 456–469. 10.1016/j.molp.2016.10.003 27746300

[B204] Xiao B. Huang Y. Tang N. Xiong L. (2007). Over-expression of a LEA gene in rice improves drought resistance under the field conditions. *Theor. Appl. Genet.* 115 35–46. 10.1007/s00122-007-0538-9 17426956

[B205] Xie K. Minkenberg B. Yang Y. (2015). Boosting CRISPR/Cas9 multiplex editing capability with the endogenous tRNA-processing system. *Proc. Natl. Acad. Sci. U.S.A.* 112 3570–3575. 10.1073/pnas.1420294112 25733849 PMC4371917

[B206] Xie K. Yang Y. (2013). RNA-guided genome editing in plants using a CRISPR/Cas system. *Mol. Plant* 6 1975–1983. 10.1093/mp/sst119 23956122

[B207] Xie Z. W. Luo M. J. Xu W. F. Chi C. W. (1997). Two reactive site locations and structure-function study of the arrowhead proteinase inhibitors, A and B, using mutagenesis. *Biochemistry* 36 5846–5852. 10.1021/bi962993c 9153425

[B208] Xue G. G. Way H. H. Richardson T. Drenth J. Joyce P. A. Mcintyre C. L. (2011). Overexpression of TaNAC69 leads to enhanced transcript levels of stress up-regulated genes and dehydration tolerance in bread wheat. *Mol. Plant* 4 697–712. 10.1093/mp/ssr013 21459832

[B209] Xue Y. Chen B. Wang R. Win A. N. Li J. Chai Y. (2018). Genome-wide survey and characterization of fatty acid desaturase gene family in Brassica napus and its parental species. *Appl. Biochem. Biotechnol.* 184 582–598. 10.1007/s12010-017-2563-8 28799009

[B210] Yan H. Q. Chang S. H. Tian Z. X. Zhang L. Sun Y. C. Li Y. (2011). Novel AroA from *Pseudomonas putida* confers tobacco plant with high tolerance to glyphosate. *PLoS One* 6:e19732. 10.1371/journal.pone.0019732 21611121 PMC3097199

[B211] Yang F. Debatosh D. Song T. Zhang J. H. (2021). Retraction note: light harvesting-like protein 3 interacts with phytoene synthase and is necessary for carotenoid and chlorophyll biosynthesis in rice. *Rice* 14:41. 10.1186/s12284-021-00484-x 33978847 PMC8116366

[B212] Yang S. Gao M. Xu C. Gao J. Deshpande S. Lin S. (2008). Alfalfa benefits from *Medicago truncatula*: the RCT1 gene from *M. truncatula* confers broad spectrum resistance to anthracnose in alfalfa. *Proc. Natl. Acad. Sci. U.S.A.* 105 12164–12169. 10.1073/pnas.0802518105 18719113 PMC2527883

[B213] Yang Y. Luang S. Harris J. Riboni M. Li Y. Bazanova N. (2018). Overexpression of the class I homeodomain transcription factor TaHDZipI-5 increases drought and frost tolerance in transgenic wheat. *Plant Biotechnol. J.* 16 1227–1240. 10.1111/pbi.12865 29193733 PMC5978581

[B214] Yang Y. Zhu G. Li R. Yan S. Fu D. Zhu B. (2017). The RNA editing factor SlORRM4 is required for normal fruit ripening in tomato. *Plant Physiol.* 175 1690–1702. 10.1104/pp.17.01265 29061908 PMC5717740

[B215] Ye X. Al-Babili S. Kloti A. Zhang J. Lucca P. Beyer P. (2000). Engineering the provitamin A (beta-carotene) biosynthetic pathway into (carotenoid-free) rice endosperm. *Science* 287 303–305. 10.1126/science.287.5451.303 10634784

[B216] Yi D. Ma L. Lin M. Li C. (2018). Development of glyphosate-resistant alfalfa (*Medicago sativa* L.) upon transformation with the GR79Ms gene encoding 5-enolpyruvylshikimate-3-phosphate synthase. *Planta* 248 211–219. 10.1007/s00425-018-2898-6 29687223

[B217] Yu F. Cao X. Liu G. Wang Q. Xia R. Zhang X. (2020). ESCRT-I component PS23A is targeted by E3 ubiquitin ligase BAT35 for proteasome-mediated degradation in modulating ABA signaling. *Mol. Plant* 13 1556–1569. 10.1016/j.molp.2020.09.008 32919085

[B218] Zaidi S. S. Tashkandi M. Mansoor S. Mahfouz M. M. (2016). Engineering plant immunity: using CRISPR/Cas9 to generate virus resistance. *Front. Plant Sci.* 7:1673. 10.3389/fpls.2016.01673 27877187 PMC5099147

[B219] Zeng Y. Wen J. Zhao W. Wang Q. Huang W. (2020). Rational improvement of rice yield and cold tolerance by editing the three genes OsPIN5b, GS3, and OsMYB30 with the CRISPR/Cas9 system. *Front. Plant Sci.* 10:1663. 10.3389/fpls.2019.01663 31993066 PMC6964726

[B220] Zhang H. Zhang J. Wei P. Zhang B. Gou F. Feng Z. (2014). The CRISPR/Cas9 system produces specific and homozygous targeted gene editing in rice in one generation. *Plant Biotechnol. J.* 12 797–807. 10.1111/pbi.12200 24854982

[B221] Zhang J. Khan S. A. Heckel D. G. Bock R. (2017). Next-generation insect-resistant plants: RNAi-mediated crop protection. *Trends Biotechnol.* 35 871–882. 10.1016/j.tibtech.2017.04.009 28822479

[B222] Zhang J. Zhou Z. Bai J. Tao X. Wang L. Zhang H. (2020). Disruption of MIR396e and MIR396f improves rice yield under nitrogen-deficient conditions. *Nat. Sci. Rev.* 7 102–112. 10.1093/nsr/nwz142 34692021 PMC8288854

[B223] Zhang L. Shen W. Fang Z. Liu B. (2016). Cry1ab/c in different stages of growth in transgenic rice Bt-shanyou63. *Front. Biosci.* 21:447–454. 10.2741/4400 26709785

[B224] Zhang X. B. Tang Q. L. Wang X. J. Wang Z. X. (2017). Development of glyphosate-tolerant transgenic cotton plants harboring the G2-aroA gene. *J. Integr. Agric.* 16 551–558. 10.1016/S2095-3119(16)61458-2

[B225] Zhang Z. Ge X. Luo X. Wang P. Fan Q. Hu G. (2018). Simultaneous editing of two copies of Gh14-3-3d confers enhanced transgene-clean plant defense against *Verticillium dahliae* in allotetraploid upland cotton. *Front. Plant Sci.* 9:842. 10.3389/fpls.2018.00842 30013582 PMC6036271

[B226] Zhao B. Lin X. Poland J. Trick H. Leach J. Hulbert S. (2005). A maize resistance gene functions against bacterial streak disease in rice. *Proc. Natl. Acad. Sci. U.S.A.* 102 15383–15388. 10.1073/pnas.0503023102 16230639 PMC1266081

[B227] Zhao Y. Zhang Z. Gao J. Wang P. Hu T. Wang Z. (2018). *Arabidopsis duodecuple* mutant of PYL ABA receptors reveals PYL repression of ABA-independent SnRK2 activity. *Cell Rep.* 23 3340–3351. 10.1016/j.celrep.2018.05.044 29898403 PMC6085104

[B228] Zhou Y. L. Xu J. L. Zhou S. C. Yu J. Xie X. W. Xu M. R. (2009). Pyramiding Xa23 and Rxo1 for resistance to two bacterial diseases into an elite indica rice variety using molecular approaches. *Mol. Breed.* 23 279–287. 10.1007/s11032-008-9232-0

[B229] Zhu J. Song N. Sun S. Yang W. Zhao H. Song W. (2016). Efficiency and inheritance of targeted mutagenesis in maize using CRISPR-Cas9. *J. Genet. Genomics* 43 25–36. 10.1016/j.jgg.2015.10.006 26842991

[B230] Zhu J. K. (2002). Salt and drought stress signal transduction in plants. *Ann. Rev. Plant Biol.* 53 247–273. 10.1146/annurev.arplant.53.091401.143329 12221975 PMC3128348

[B231] Zhu J. K. (2016). Abiotic stress signaling and responses in plants. *Cell* 167 313–324. 10.1016/j.cell.2016.08.029 27716505 PMC5104190

